# Phytotoxic Ozone Dose–Response Relationships for Durum Wheat (*Triticum durum*, Desf.)

**DOI:** 10.3390/plants13050573

**Published:** 2024-02-20

**Authors:** Riccardo Marzuoli, Franco Faoro, Valentina Picchi, Giacomo A. Gerosa

**Affiliations:** 1 Department of Mathematics and Physics, Catholic University of the Sacred Heart, Via Garzetta 48, 25133 Brescia, Italy; giacomo.gerosa@unicatt.it; 2 CREA Research Centre for Engineering and Agro-Food Processing, Via Venezian 26, 20133 Milano, Italy; franco.faoro@unimi.it (F.F.); valentina.picchi@crea.gov.it (V.P.)

**Keywords:** durum wheat, phytotoxic ozone dose, open-top chambers, crop yield

## Abstract

Ozone (O_3_) pollution poses a significant threat to global crop productivity, particularly for wheat, one of the most important staple foods. While bread wheat (*Triticum aestivum*) is unequivocally considered highly sensitive to O_3_, durum wheat (*Triticum durum*) was often found to be more tolerant. This study investigated the O_3_ dose–response relationships for durum wheat in the Mediterranean region, focusing mainly on grain yield losses, and utilizing the phytotoxic ozone dose (POD) metric to describe the intensity of the stressor. The results from two experiments with Open-Top Chambers performed in 2013 and 2014 on two relatively sensitive durum wheat cultivars confirmed that this wheat species is far more tolerant than bread wheat. The use of a local parameterization of a stomatal conductance model based on field measurements did not significantly improve the dose–response relationships obtained in comparison to the generic parameterization suggested by the Mapping Manual of the United Nations Economic Commission for Europe (UNECE). The POD6 critical level of 5 mmolO_3_ m^−2^ for 5% grain yield loss was remarkably higher than the one established for bread wheat with analogous experiments, highlighting that O_3_ risk assessments based on bread wheat may largely overestimate the damage in the Mediterranean region where durum wheat cultivation prevails.

## 1. Introduction

Ozone (O_3_) is a highly reactive air pollutant of secondary formation that can induce a range of physiological and biochemical alterations in plants, such as oxidative stress, cell membrane damage, and reduced photosynthesis [1]. For this reason, tropospheric O_3_ pollution poses a significant threat to crop productivity and food security worldwide and its rising ground-level concentrations have become a major environmental issue, with detrimental consequences for agriculture, crop yields, and crop quality [2,3].

Among the numerous crops affected, wheat is of particular concern due to its economic importance as one of the world’s most important staple food crops [4] and its susceptibility to O_3_-induced phytotoxicity [5,6,7]. Although wheat is considered an O_3_-sensitive crop, different species and cultivars exhibit a wide range of ozone sensitivity [8,9,10,11,12,13]. An interesting observation in this regard is the contrast in sensitivity between bread wheat (*Triticum aestivum*), which is considered highly sensitive [14,15], and durum wheat (*Triticum durum*), apparently a more tolerant species, which however often shows severe visible symptoms [16]. This discrepancy can be attributed to different factors such as the studied cultivar, agricultural practices, geographical location, and meteorological conditions during the growing season [17,18]. The complexity of crop responses to O_3_ needs an in-depth exploration to elucidate the precise dynamics underlying O_3_-induced phytotoxicity. The evaluation of crop sensitivity to O_3_ has traditionally focused on two primary indicators: crop yield (or plant growth) decline and the emergence of O_3_-induced foliar injuries [19,20,21,22,23]. It has been widely recognized that the manifestation of these adverse effects correlates more closely with the cumulative O_3_ uptake absorbed by plant leaves through stomata rather than with simple exposure to ambient O_3_ levels [15,24,25].

The impact of O_3_ pollution extends not only to crops but also to human health and terrestrial ecosystem integrity in Europe. This concern led to the establishment of the Convention on Long-Range Transboundary Air Pollution (LRTAP), managed by the UNECE, which seeks to formulate policies aimed at reducing tropospheric O_3_ concentrations and facilitates international cooperation for the collection of data and scientific insights. The O_3_ risk assessment methodology used within the LRTAP Convention is based on the estimation of the O_3_ exceedances of critical levels. These critical levels are determined by defining concentrations, cumulative exposures (AOT40, Accumulated Ozone over a Threshold of 40 ppb), or cumulative stomatal fluxes (or POD, phytotoxic ozone dose) above which direct adverse effects on sensitive vegetation may occur [26].

Critical level values based on exposure-response relationships are mainly derived from experiments with controlled conditions (Open-Top Chambers or fumigation chambers) and do not take into account the stomatal influence on the amount of O_3_ entering the plant. Indeed, evidence from several studies indicates that methodologies based on stomatal O_3_ uptake are more accurate in assessing the effects of O_3_ on vegetation because they consider both atmospheric chemistry and plant physiology, which are the two main key factors affecting plant response to this atmospheric pollutant [27,28].

This approach requires the modeling of stomatal conductance for the quantification of the POD above a threshold *Y*, which represents the stomatal O_3_ flux exceeding a value Y which represents the amount of the O_3_ stomatal flux instantaneously detoxified by the plant’s defense systems. For wheat, a threshold of 6 nmol m^−2^ s^−1^ (POD6) was proposed according to several experiments conducted in controlled conditions [26,28].

Moreover, there is growing concern that dose–effect relationships derived from experiments performed in Central and Northern European conditions may overestimate ozone’s impact in Southern European regions as climatic conditions in the Mediterranean area are strongly different, showing higher temperatures and vapor pressure deficit (VPD) conditions, lower rain regimes, and severe summer drought episodes [29,30,31]. In this regard, it has been recently suggested that the impact of O_3_ on agronomic yield should be quantified using dose–response relationships for each cultivation area, taking into account the effects of seasonal change in O_3_ concentrations, the cultivars, and the climate [5]. To date, however, no dose–response relationship has ever been reported in the literature for grain yield loss of durum wheat.

In light of these challenges, the primary aim of this study was to derive a dose–response relationship for *T*. *durum*, utilizing the POD6 metric and the relative grain yield losses observed in experiments conducted in Southern Europe. The base-line hypotheses of this work were that *T*. *durum* growing in Mediterranean conditions is more tolerant to O_3_ than *T*. *aestivum* in the same conditions and that the use of a local parameterization of *g_s_* for the estimation of POD6 critical levels provides more reliable results in terms of statistical significance of the dose–response relationships than the generic parameterization proposed by the UNECE for the Mediterranean region [26]. Moreover, the results from this investigation could be important to enhance the accuracy of local and large-scale models currently employed to estimate the impact of O_3_ on vegetation at local and regional levels.

## 2. Results

### 2.1. Meteoclimatic Conditions during the Experiments

**Figure 1:**
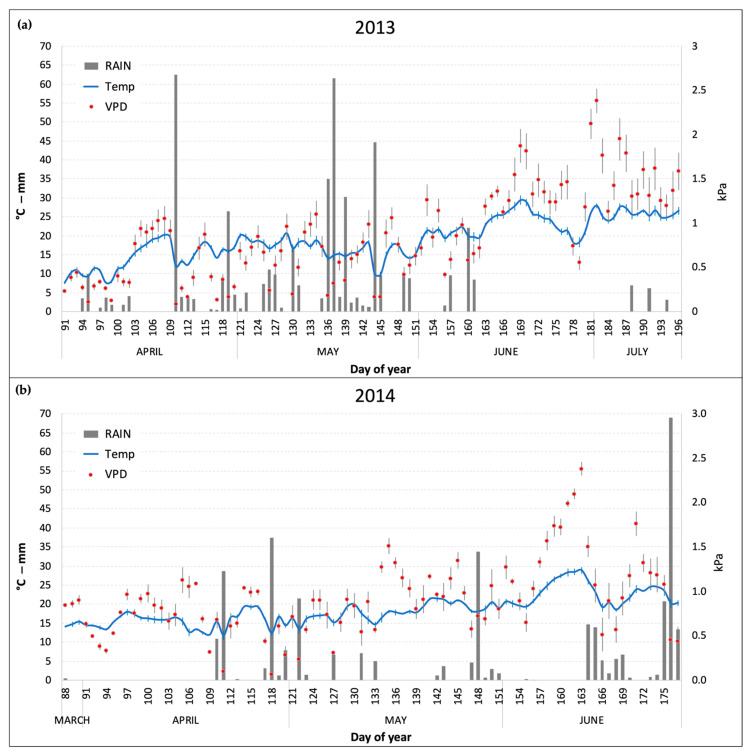
Daily average of temperature (Temp) and vapor pressure deficit (VPD) and daily sum of rain inside the OTCs during the two experimental periods. (**a**) 2013 experiment; (**b**) 2014 experiment. Bars for Temp and VPD series represent the standard error of the mean.

The daily mean values of the main meteoclimatic conditions (temperature, precipitation, and VPD) that were monitored inside the OTCs during the two experiments are reported in Figure 1. April and May were warmer in 2014 compared to 2013, which was characterized by an unusually cold spring (especially in the first two weeks of April) and frequent precipitations until the end of May (141 and 276 mm in April and May, respectively). In 2014, conversely (Figure 1b), precipitations were mainly concentrated in short periods at the end of April and May and in the last two weeks of June, before the harvest (June showed a total rain of 154 mm). VPD levels were on average higher in 2014 (due to lower precipitations) with peaks well above 1.5 kPa (as daily average) in the days between the end of May and the beginning of June. Nonetheless, in 2013 July recorded the highest VPD monthly average of the two growing seasons (1.53 kPa) due to a prolonged period without precipitations and high temperatures (Figure 1a). 

### 2.2. The Stomatal Conductance Model of T. durum Based on Local Parameterization

A dataset of around 900 measurements (200 in 2013, and 700 in 2014) of adaxial surface stomatal conductance (*g_s_*) measurements was used to draw a local parameterization of the stomatal conductance of *T*. *durum* (Table 1). The maximum stomatal conductance (*g_max_*) to water was determined as the 90th percentile of all valid *g_s_* measurements, regardless of the cultivar and measurement year considered. This value (700 mmolH_2_O m^−2^ s^−1^ per Projected Leaf Area, PLA) was then multiplied by the diffusivity ratio coefficient (0.663) to obtain a maximum stomatal conductance to O_3_ of 465 mmolO_3_ m^−2^ s^−1^ PLA. The local *g_s_* measurements yielded a higher value of *g_max_* compared to the value reported in the Mapping Manual (MM) of the LRTAP Convention (Table 1, 410 mmolO_3_ m^−2^ s^−1^ PLA), which sets the standard methodology for POD calculation.

Table 1 reports also the different values of the coefficients for the limiting functions *f*_Temp_, *f*_Light_, *f*_VPD,_ and *f*_SWC_, calculated from the local *g_s_* measurements in comparison to those reported for the MM parameterization of *g_s_* for *T*. *durum*. The limiting functions, which give a value between 0 and 1, were defined with a boundary layer analysis based on the values corresponding to the 98th percentile of the relative stomatal conductance (*g_rel_* = stomatal conductance value relative to *g_max_*) plotted versus each class of values of the environmental variable considered. It was thus possible to make the *g_s_* model less dependent on the outliers representing measurements of doubtful quality. The *f*_Phen_ function based on the MM parameterization, which accounts for phenological stages limitation on *g_s_*, was also used for the local parameterization because we did not collect evidence of significant differences.

**Figure 2:**
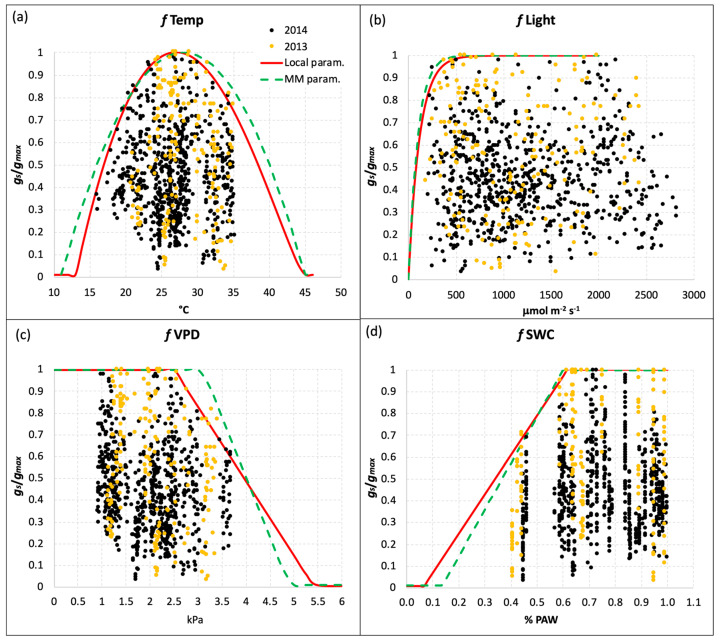
Limiting functions for *g_s_* due to (**a**) temperature, (**b**) PAR, (**c**) VPD, and (**d**) SWC for the local parameterization (red line) defined with local *g_s_* measurements, and the generic parameterization reported by the Mapping Manual (green dotted line) for durum wheat. Yellow and black dots represent the *g_s_* measurements performed in 2013 and 2014, respectively.

Figure 2 reports the results of the boundary layer analysis for the four limiting functions derived from the local *g_s_* measurements. The red lines define the local limiting function for each environmental variable (Temp, Light, VPD, and SWC), while the green dotted lines represent the generic parameterization of the MM. In general, the two parameterizations were quite similar, with very slight differences for *f*_Temp_ and *f*_Light_ (Figure 2a,b), which resulted in more restrictions in the local parameterization. The limiting function for SWC (Figure 2d) for the local parameterization was less steep than the generic one, with a slightly higher threshold of Plant Available Water (PAW) below which the function starts to decrease (19% for the local parameterization vs 18% for the MM one), and a lower value of PAW corresponding to the function reaching the minimum value (*f*_min_ = 0.01, Table 1). Besides the *g_max_* value, the most evident difference between the local and generic parameterization was found for the *f*_VPD_ limiting function (Figure 2c): the VPD threshold above which the function (and the relative *g_s_*) starts to decrease was 2.5 kPa, while for the generic parameterization this value was 25% higher (3.1 kPa). This feature resulted in a less steep limiting function for the local parameterization, which reaches the minimum value at VPD = 5.4 kPa. This is well above the value of 4.9 kPa reported in the MM for the generic parameterization (Figure 2c and Table 2).

### 2.3. Phytotoxic Ozone Dose Absorbed by Plants during the Experiments

The POD absorbed by T. durum plants was calculated as the POD6 index, which accounts for a detoxification threshold of 6 nmolO_3_ m^−2^ PLA s^−1^ as recommended for wheat by the MM [26]. Thanks to the availability of a generic and local parameterization for *g_s_*, we performed the calculation of the POD6 for each combination of experiment year, *g_s_* model parameterization, and O_3_ treatment. Moreover, the calculation of POD6 also followed a cultivar-specific pattern because of some differences between Colombo and Sculptur in their phenological development. Table 2 reports the starting date of the main phenological stages of the two cultivars in 2013 and 2014, together with information on the O_3_ application time. 

**Figure 3:**
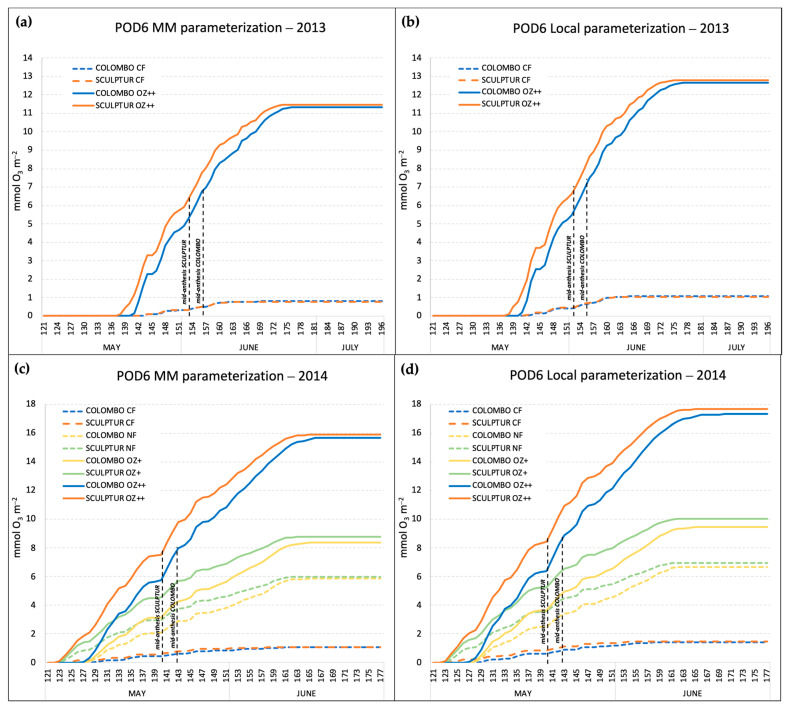
Seasonal accumulation of the POD6 index in the different O_3_ treatments, calculated with the Mapping Manual *g_s_* parameterization in 2013 (**a**) and 2014 (**c**), and with the local *g_s_* parameterization in 2013 (**b**) and 2014 (**d**). Dashed lines indicate the mid-anthesis dates for the two durum wheat cultivars.

In both experiments, Sculptur entered the anthesis stage 3 days before Colombo (Table 2), and this affected the POD6 accumulation period which is based on the thermal sum value (expressed in °C day, Table 1) calculated backward and onwards from the mid-anthesis date. The accumulation patterns of the POD6 in 2013 and 2014, according to the different O_3_ treatments, cultivars, and *g_s_* model parameterizations, are shown in Figure 3.

The POD6 accumulation started around two weeks earlier in 2014 compared to 2013 (122 DAS in 2014, 136 DAS in 2013), and this was mainly due to warmer conditions in April and May 2014 that accelerated the phenological development (anthesis was reached one week earlier in 2014 than in 2013) and to an earlier start in the O_3_ fumigation treatments (Table 2).

The comparison between POD6 calculated with the MM *g_s_* model parameterization and with the local *g_s_* model parameterization showed that the local parameterization resulted in the absorption of a much higher O_3_ dose. In 2014, the local parameterization gave POD6 values of 17.68 and 17.31 mmolO_3_ m^−2^ for Sculptur and Colombo, respectively, for the plants in the OZ++ treatments, while with the MM parameterization, those values were around 10% lower (15.90 and 15.68 mmolO_3_ m^−2^ for Sculptur and Colombo, respectively). Considering that the local parameterization was based on a *g_max_* value for *T. durum* that was 13% higher than the *g_max_* used for the MM parameterization (465 vs. 410 nmolO_3_ m^−2^ s^−1^, Table 1), the difference can be mainly explained by the higher rate of O_3_ uptake estimated with the local *g_s_* model. In 2013, the difference between the local and the MM parameterization in the OZ++ plants was almost the same (−10.4%), although the absolute values of POD6 were generally lower than in 2014 because of a slightly lower O_3_ treatment applied in the OZ++ OTCs (+50% of O_3_ in 2013, +60% in 2014). In 2014, plants of the CF-OTCs experienced POD6 values around 1.52 and 1.14 mmolO_3_ m^−2^ with the local and the MM parameterization, respectively. In 2013, the CF treatments gave POD6 values of 1.07 and 0.78 mmolO_3_ m^−2^. 

In the 2014 experiment, a couple of intermediate O_3_ treatments (NF and OZ+) were also applied, and the final POD6 values obtained with the local parameterization of *g_s_* were on average 6.82 and 9.74 mmolO_3_ m^−2^. In the same treatments, with the MM parameterization, the POD6 values were 13.2 and 11.9% lower. The final values of POD6 for the different combinations of year, cultivar, O_3_ treatment, and *g_s_* model parameterization are reported in Table 3. For completeness, Table 3 reports also the AOT40 values (period April–June) registered for each O_3_ treatment applied in 2013 and 2014. In both years, the accumulated O_3_ exposure in the control treatments CF was well below the AOT40 critical level set by UNECE for agricultural crop protection (3 ppm.h), and by contrast, it was more than seven times above the critical level in the most ozonated treatments (OZ++ OTCs).

### 2.4. Crop Yield

**Figure 4:**
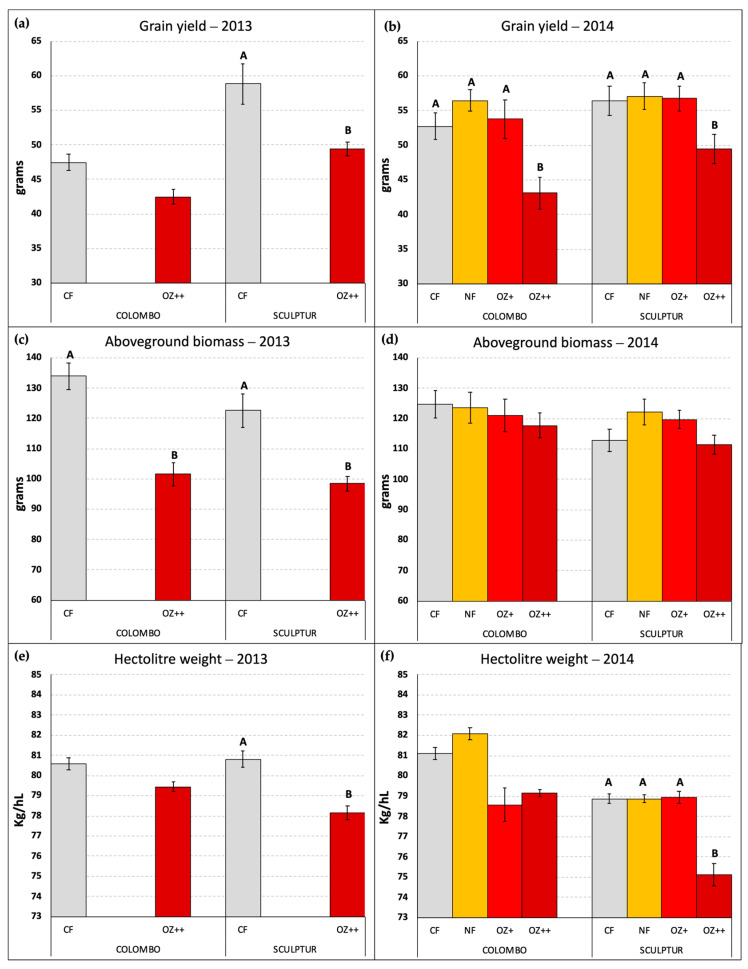
Grain yield per pot (**a**,**b**), aboveground dry biomass per pot (**c**,**d**), and hectolitre weight per OTC (**e**,**f**) measured at the end of the two experiments in 2013 and 2014, in the different O_3_ treatments. Different letters indicate a statistically significant difference assessed with the ANOVA test and the Tukey post hoc test. Bars represent the standard error of the mean. Data in (**a**,**c**) graphs were already published in Monga et al. [9].

Figure 4 reports the grain yield and the amount of aboveground dry biomass per pot together with the hectolitre weight measured for the grains obtained in each O_3_ treatment applied. Only the pots containing three fully developed plants of *T*. *durum* were considered in this elaboration. In 2014, three pots of Colombo and one pot of Sculptur plants were excluded because of a lack of one or more plants, while in 2013 all the pots were included in the measurements.

In 2013, the difference in grain yield between CF and OZ++ plants was statistically significant only for the Sculptur cultivar (−16.0%, *p* = 0.040), although a clear decrease in grain yield was also evident for Colombo (−10.5%, *p* = 0.234, Figure 4a). In 2014, the decrease in grain yield due to the O_3_ treatments was statistically significant for both cultivars, although only OZ++ plants showed a significant decrease (−18.2% *p* = 0.000 in Colombo, −10.9% *p* = 0.027 in Sculptur) compared to the CF treatments. The intermediate O_3_ treatments (NF and OZ+) did not show significant grain yield losses compared to the CF plants (Figure 4b).

The total aboveground dry biomass of plants was lowered by the highest O_3_ treatment only in 2013 on both cultivars, showing a −24.3% decrease in Colombo (*p* = 0.004) and a −19.7% decrease in Sculptur (*p* = 0.018, Figure 4c). This result was not replicated in 2014 when the aboveground dry biomass was not statistically affected by O_3_ and only the cultivar Colombo showed a −5.5% decrease in the OZ++ plants (Figure 4d).

Contrasting results on the effect of O_3_ on other crop-yield parameters (data not shown) were found for the harvest index (significantly decreased in 2013 in Colombo, but not in 2014), the stems’ dry biomass (significantly decreased in both cultivars in 2013, but not in 2014) and the number of spikes produced (significantly decreased in Sculptur in 2013, but not in 2014).

Regarding the hectolitre weight, which can be considered as a grain quality indicator, the results obtained in 2014 confirmed what was already observed in 2013 (Figure 4e,f). Sculptur showed a statistically significant decrease in the OZ++ treatment (−3.2% *p* = 0.038 and −4.1% *p* = 0.000 in 2013 and 2014, respectively), while Colombo plants were not statistically affected, although in both years the values tended to decrease in O_3_ treated plants (−1.4% and −2.4% in 2013 and 2014, respectively, for the OZ++ treatment). Also, in this case, the intermediate O_3_ treatments applied in 2014 did not show any particularly different behavior compared to CF plants, with the sole exception of Colombo OZ+ plants that showed a marked decrease in the hectolitre weight, even greater than the OZ++ plants (−3.1%, Figure 4f).

### 2.5. Dose–Response Relationships for Durum Wheat

The data collected in the two OTC experiments on grain yield, aboveground biomass, and grain hectolitre weight were used to derive dose–response relationships based on the POD6 values calculated for each O_3_ treatment, separately for each cultivar in the two years of experimentation. For the linear regression between these values and the relative yield values of the considered agronomic parameter, the data were grouped together because the purpose of the study was to derive a single dose–response relationship for the *T. durum* species, not for individual cultivars.

**Figure 5:**
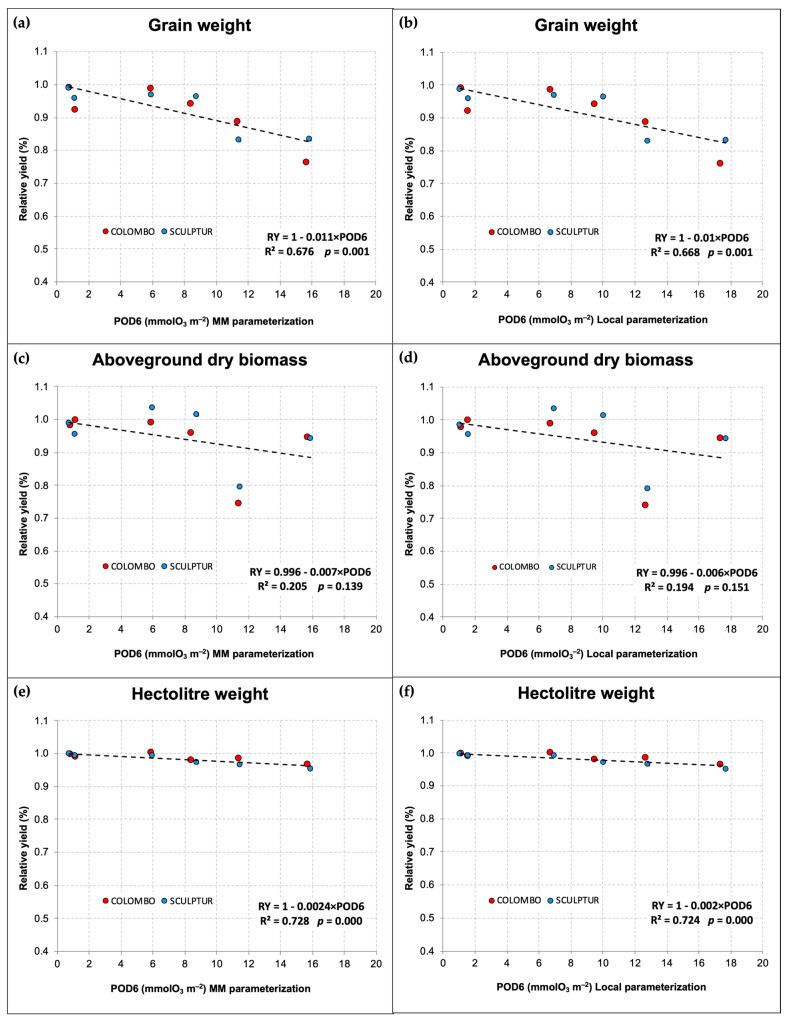
Dose–response relationships (dashed lines) for durum wheat based on POD6 and crop-yield parameters, calculated with the MM *g_s_* parameterization (**a**,**c**,**e**) and with the local *g_s_* parameterization (**b**,**d**,**f**). The regression equations and statistical significance of the dose–response relationships are reported in each graph.

As the use of the *g_s_* parameterization based on the MM and that based on local measurements led to different calculations of POD6 values, we present in Figure 5 the comparison between the dose–response relationships obtained with the two different approaches. The relationships show only a slight difference in the predicted damage intensity for each unit of POD6 absorbed by the plants during the season. Both parameterizations lead to the definition of statistically significant dose–response relationships for grain yield and hectolitre weight with *p* ≤ 0.001, while for the aboveground dry biomass, in both cases, the relationship did not show statistical significance (*p* > 0.05).

In the case of grain yield, the use of the local *g_s_* parameterization leads to a dose–response relationship predicting a relative loss of −1% for each unit of POD6 absorbed during the accumulation period (Figure 5b). With the MM parameterization, however, the predicted loss was slightly higher, −1.1% for each unit of POD6. The same situation applies to the relative decrease in hectolitre weight, which was −0.20% and −0.24%, respectively, for the relationship based on local parameterization and on the MM parameterization.

## 3. Discussion

This study represents the first attempt to define a dose–response relationship based on the POD6 index and the relative decrease in grain yield for durum wheat. Within the framework of the UNECE-LRTAP, similar dose–response relationships have been defined only for *T*. *aestivum* among the cereal crops and were based on OTC experiments conducted mainly in Northern Europe, often in environmental conditions very different from those of Mediterranean Europe. One of the initial hypotheses was that the *T*. *durum* wheat species, which is predominantly cultivated in Southern Europe and in the Mediterranean area, might be more O_3_-tolerant than *T*. *aestivum* because of a better adaptation to the conditions of high temperatures and high O_3_ levels typical of the Mediterranean environment. Our observations seem to confirm the above-mentioned hypothesis and the findings from the relatively limited studies conducted on the agronomic response of durum wheat to O_3_. For instance, Biswas et al. [22] highlighted that *T*. *turgidum* spp. *durum* was the most O_3_-tolerant wheat species compared to other tested species (*T*. *aestivum* and *T*. *monococcum*), even though it still exhibited reductions in agronomic yield. Similarly, Tomer et al. [32] reported greater O_3_ tolerance in *T*. *durum* compared to *T*. *aestivum* in a two-year OTC experiment performed in India. Additionally, Gerosa et al. [33] found that two durum wheat cultivars (Virgilio and Neodur) that were exposed to a combination of high O_3_ levels and saline irrigation in an OTC experiment showed no reduction in the main agronomic parameters when the plants were exposed only to increased O_3_ levels. Nevertheless, other studies have identified O_3_-sensitive cultivars in relation to biomass growth and grain production [34,35], demonstrating that, similarly to *T*. *aestivum*, there is a remarkable variability in the O_3_-induced response within the *Triticum* genus depending on the species and cultivar considered [36]. 

The results produced by our experiments and those already published by Monga et al. [9] and Picchi et al. [37] pointed in the same direction. In particular, Monga et al. [9], in their varietal screening conducted in 2013, found that only one cultivar (Sculptur) out of the five tested showed a significant decrease in grain yield due to high O_3_ levels, and only two (Colombo and Sculptur) out of five showed a significant decrease in the aboveground dry biomass. In their 2014 experiment, Picchi et al. [37] focused on the two aforementioned cultivars, namely the most sensitive to O_3_ among those tested, showing that they exhibited opposing behaviors in terms of visible foliar symptoms, with Colombo being symptomatic and Sculptur being symptomless. Their findings indicated that this difference could be attributed to the higher constitutive pool of antioxidants in Sculptur that effectively thwarted the initiation of leaf symptoms and the collapse of mesophyll cells.

In this study, we instead focused on the agronomic yield of the two most sensitive cultivars to define critical levels for crop protection based on the metric of the phytotoxic ozone dose (POD), now recognized as the most suitable metric for O_3_ risk assessment to estimate damage to plants’ productivity. Despite the different responses in terms of visible foliar symptoms on the flag leaf, both cultivars in 2013 and 2014 showed a decrease in grain yield and hectolitre weight, confirming that, even for agricultural plants, the reduction in growth rate and productivity is not always accompanied by the onset of visible foliar symptoms [16,38], as already highlighted for forest species [39]. The results show that a significant reduction in grain yield and hectolitre weight in 2014 was observed exclusively in plants exposed to the highest O_3_ treatment (Figure 4b,f, OZ++ treatments), except for Colombo, which exhibited a decrease in hectolitre weight even in the intermediate OZ+ treatment (Figure 4f), although not significant. Under control conditions (−50% of the ambient O_3_ concentration, CF-OTCs), Sculptur proved to be more productive compared to Colombo (+24% and +7%, in 2013 and 2014, respectively), although this difference was remarkably smaller in 2014 compared to the experiment conducted in 2013. Despite the consistency of this result, Sculptur plants in 2014 did not show the same loss in grain yield observed in the previous experiment (−16% in 2013 vs. −11% in 2014, Figure 4a,b), and moreover the decrease in relative yield in 2014 was not statistically significant, probably because the statistical analysis was performed taking into account also the intermediate O_3_ treatments (NF and OZ+) that were not applied in 2013. Colombo plants under OZ++ conditions instead showed a higher grain yield loss in 2014 than in 2013 (−18% vs. −10%) confirming that both cultivars showed a similar sensitivity to O_3_. Moreover, in 2014 the total aboveground biomass of both cultivars plants remained almost unaffected by ozone (−5% and −1% in Colombo and Sculptur, respectively), indicating a clear shift in the allocation and partition of the photosynthesis products from the grain to the stems biomass, with a consequent decrease in the harvest index (−14% and −10% in Colombo and Sculptur, respectively). This outcome was also found in spring wheat by Pleijel et al. [8], who reported a significant reduction in the grain yield and harvest index in non-filtered OTCs with the addition of 40 ppb of O_3_, and by Rai et al. [40], who reported a reduction in both harvest index and hectolitre weight of 20.7% and 4.8%, respectively, for wheat grown in non-filtered OTCs compared to filtered OTCs. However, in our case, this result must be further investigated since it is not confirmed by the 2013 experiment which presented an opposite situation [9].

The fact that in 2014, plants exposed to intermediate O_3_ treatments (NF and OZ+) did not show significant negative effects on the agronomic parameters suggests a certain tolerance of *T*. *durum* to O_3_, likely linked to its adaptation to drier and warmer climates, which often promote the formation of high O_3_ levels during the wheat-growing season. Our study, nonetheless, involved two O_3_-sensitive cultivars (selected through varietal screening) with the aim of defining precautionary O_3_ critical levels that ensure protection not only for the sensitive cultivars included in the study but also for other varieties of durum wheat. Currently, the O_3_ critical level for *T*. *durum* within the UNECE framework is still undefined, and the application of the critical level established for *T*. *aestivum* (1.3 mmolO_3_ m^−2^ PLA) to all the wheat species could lead to significant overestimations of O_3_ damage in durum wheat, particularly in Southern European countries where durum wheat is largely cultivated [41], as for example in Italy where *T*. *durum* coverage was 2.4 times larger than *T*. *aestivum* in the last decade [42].

The use of a local *g_s_* model, based on a comprehensive dataset of field measurements on the flag leaf in both wheat growing seasons, allowed for a comparison between the POD6 calculated with this *g_s_* model and the same index calculated with the generic *g_s_* model for the Mediterranean region available in the MM [26]. The local parameterization did not lead to a significant improvement in the dose–response relationships compared to those defined with the MM parameterization (Figure 5). The latter was essentially provided by the work of Gonzalez-Fernandez et al. [31] and based on datasets of *g_s_* measurements conducted in central Spain between 1998 and 2002, and between 2009 and 2010. The comparison between the two different parameterizations highlights only minor differences in the limiting functions outlined, with *f*_Temp_ and *f*_PAR_ slightly more restrictive in the local parameterization, and *f*_VPD_ beginning to exert a limitation on *g_max_* starting from a threshold (VPD_max_) of 2.5 kPa (Table 1), a value that is about 20% lower than the threshold proposed in the MM (3.1 kPa). The same limiting function, however, presented a higher VPD_min_ value (5.4 kPa vs. 4.9 kPa), which determines a lower limitation at very high levels of air VPD. The major difference between the two approaches was found in the value of *g_max_*, which is also a key parameter in determining the calculation of the phytotoxic ozone dose [43]. In the local parameterization, *g_max_* was 13% higher than the value proposed in the MM (410 mmolO_3_ m^−2^ s^−1^ PLA) from the study of Gonzalez-Fernandez et al. [31]. However, this latter value was calculated as the mean value of *g_max_* from two datasets that provided *g_max_* values of 362 and 459 mmolO_3_ m^−2^ s^−1^, respectively. Therefore, our value (465 mmolO_3_ m^−2^ s^−1^) was substantially in line with what was reported by Gonzalez-Fernandez et al. [31], especially considering that *g_s_* measurements in our experiments were conducted on irrigated plants and thus under less limiting conditions compared to those conducted in Spain on rainfed crops. Given the small differences in the limiting functions of the two compared models, it does not seem necessary to modify the parameterization of the MM for *T*. *durum*, except for a slight adjustment of the *g_max_* value that could lead to better estimates of the POD6 for the O_3_ risk assessments of this crop.

The POD6–response relationships, outlined with data collected in both experiments and grouping the two cultivars, were significant for the grain yield loss and the hectolitre weight decrease. Specifically, the relationships showed very similar coefficients of determination (R^2^) for both parameterizations used (R^2^ = 0.676 and 0.668 for grain yield loss using the MM and the local parameterization, respectively). The use of a *g_s_* local parameterization did not result in statistically better dose–response relationships. Also, for hectolitre weight reduction, in both POD6 calculation contexts, linear regression was highly significant with almost identical R^2^ values. However, the local parameterization, being based on a *g_max_* calculated from a large dataset of field measurements, can be considered more reliable in establishing the critical level of POD6 to mitigate grain yield reduction. In this case, if we consider as acceptable a relative grain yield loss not exceeding 5% compared to a hypothetical null POD6 situation (see Section 2.5), the identified critical level for POD6 is 5 mmolO_3_ m^−2^ PLA, while with the MM parameterization, the same critical level drops to 4.5 mmolO_3_ m^−2^ PLA. In both cases, however, the identified critical level is almost four times higher than the critical level established for *T*. *aestivum* [26], confirming that, within the *Triticum* genus, *T*. *durum* is to be considered more O_3_-tolerant than *T*. *aestivum*, even when considering cultivars more susceptible to damage in terms of agronomic yield. Regarding hectolitre weight, which is an important parameter for evaluating grain quality and milling yield for flour production, the POD6 critical level is 5 times higher (25 mmolO_3_ m^−2^ PLA) with the local *g_s_* parameterization and 4.6 times higher with the MM parameterization (20.8 mmolO_3_ m^−2^ PLA). However, if we consider the absolute values of hectolitre weight recorded during the two experiments for each cultivar, and classify the grain quality according to the official standards UNI 10709 [44], then we could establish a critical level of POD6 for grain quality around 4 mmolO_3_ m^−2^ PLA for a drop below the first-class quality (hectolitre weight < 80 kg/hL) and between 14 and 15 mmolO_3_ m^−2^ PLA for a drop to the lowest class quality (hectolitre weight < 78 kg/hL).

Concerning the aboveground biomass, the contrasting results obtained in the two experiments did not allow for the definition of a statistically significant dose–response relationship, and the overall effect of O_3_ and POD6 on this variable remains uncertain. The data in Figure 5c,d reveal a misalignment of aboveground biomass values for the 2013 experiment, which were markedly lower than those of 2014. Unlike in 2013, in 2014 the effect of O_3_ on aboveground biomass was not significant (Figure 4d), and in Sculptur plants, we even observed a slight, although not significant, hormetic response to increased O_3_ levels in the intermediate treatments. This kind of response was also detected in both cultivars for the grain yield data, which was inconsistent with the O_3_ gradient applied. Unexpectedly, despite the lower O_3_ level, plants in CF-OTCs showed a lower grain yield compared to the plants in NF and OZ+ OTCs. This result could lead to important biases in the assessment of the relative effect of O_3_ when we use a linear regression to define the reference yield at hypothetical null POD6. Nevertheless, hormetic responses in some growth parameters were also observed in previous OTC experiments on deciduous oak plants exposed to different levels of O_3_ [45], and the authors supposed that this could be related to some sort of fertilization effect on the plants belonging to OTCs without charcoal filters, due to higher levels of NO concentrations. Analogous observations of lower NO concentrations in CF-OTCs were also reported by Pleijel et al. [46] and by Olszyk et al. [47] even if no hormetic response to O_3_ was observed in their experiments. This hypothesis, therefore, remains largely unproven and requires further investigation. The hormetic response to O_3,_ moreover, could be simply explained as a typical bi-phasic/non-linear reaction to an abiotic stress, which is considered very common in plant stress biology. Some authors, in fact, have hypothesized that in the O_3_ stress physiology, major stress components such as Reactive Oxygen Species that are eventually involved in the O_3_ stress response show typical biphasic-hormetic dose–time response patterns [48].

In conclusion, the dose–response relationship for relative grain yield defined in this study can be considered as a first step towards the correct quantification of O_3_-induced yield losses in durum wheat for the Mediterranean area risk assessments. Beyond some limitations of our study related to the use of pots and irrigation that could possibly affect the responses, it is important to underline that validation tests with field-grown durum wheat are still recommended before new critical levels for this wheat species can be adopted.

## 4. Materials and Methods

### 4.1. Open-Top Chambers Experiments

Two Open-Top Chambers (OTCs) experiments were performed in 2013 and 2014 in the OTC facility of Curno (Bergamo, Italy Lat. 45°41′17″ N, Long. 9°36′40″ E, elev. 242 m a.s.l.). The first experiment was a varietal screening on potted plants of five different *T*. *durum* cultivars performed between March and July 2013, while the second experiment was an investigation on the efficiency of chitosan as an O_3_ protectant for *T*. *durum* performed between March and July 2014 [37]. The 2013 experiment investigated five cultivars of *T*. *durum* (Colombo, Gallareta, Pharaon, Sculptur, and Vitron) which were grown in 4 OTCs subdivided into two treatments (CF: ambient air concentration reduced at 50% and OZ++: ambient air concentration implemented with +50% of O_3_). Plants were placed in the OTCs on 2 April 2013 and the O_3_ treatments were performed daily (9.00 AM–5.00 PM) starting from 6th May (just before the emergence of flag leaves) until 30th June, for a total of 56 days of treatment. The details on the methodology and some results of this experiment can be found in Monga et al. [9]. In the 2014 experiment, potted plants of the two most O_3_-sensitive cultivars that emerged from the previous experiment (in terms of grain relative yield and biomass) were grown in 12 OTCs subdivided into 4 treatments (CF:O_3_ filtered at −50% compared to ambient concentration, NF: non-filtered air, OZ+:O_3_ ambient concentration incremented by 30%, and OZ++:O_3_ ambient concentration incremented by 60%). In this experiment, the O_3_ treatments were applied again daily (9.00 AM–5.00 PM) starting from 23 April until 24 June for a total of 63 days of treatment (Table 1). During the experiment, plants were also subject to a sub-treatment with chitosan at 3 levels (CTRL: tap water, chitosan with 40 kDa molecular weight, and chitosan with 300 kDa molecular weight). A detailed methodology and some results of this experiment can be found in Picchi et al. [37]. For this analysis, we extrapolated only the data relative to the two common cultivars for both experiments (Colombo and Sculptur) and to the control plants of the chitosan treatment of the second experiment (i.e., plants that were not treated with chitosan). The O_3_ concentration during both experiments was continuously monitored at 1 m height using an O_3_ analyzer 1108 RS (DASIBI, Milan, Italy) through a solenoid valve system that provided the switching from one to the other OTCs. Agrometeorological parameters such as PAR (photosynthetically active radiation), air temperature, relative humidity, and rainfall were also measured within the OTCs (see details in [9]). Plants were automatically irrigated at night in order to avoid water stress and keep soil water content close to field capacity. Soil water content in the pots was monitored with two EC5 Time Domain Reflectometers (Decagon Devices, Pullman, WA, USA).

### 4.2. Ozone Metrics Calculation

The O_3_ stomatal dose received by plants was calculated by applying a multiplicative model for stomatal conductance (Jarvis, 1976) and a big-leaf O_3_ deposition scheme, following the methodology developed within the LRTAP convention and described in Chapter 3 of the Mapping Manual for critical levels exceedances calculation [26].

(1) $$g_{s}=g_{max}\times f_{Phen}\times f_{Light}\times max\left\{f_{min},\;(f_{Temp}\times f_{VPD}\times f_{SWC})\}\right.$$

where *g_s_* is the actual stomatal conductance (mmolH_2_O m^−2^ s^− 1^ PLA) and *g_max_* is the species-specific maximum stomatal conductance to water (mmolH_2_O m^−2^ s^−1^ PLA). The *f* functions (all ranging between 0 and 1) describe the relative effects of the phenology and of the different environmental conditions (temperature, vapor pressure deficit (VPD), and soil water content (SWC)) on *g_max_*, to calculate the actual stomatal conductance *g_s_*. The *f*_min_ represents the minimum stomatal conductance expressed relatively to *g_max_*. 

The parameterization of the stomatal conductance model was based on the values reported in the MM (LRTAP, 2017) for *T*. *durum* in Mediterranean conditions. The *g_max_* value for *T*. *durum* was assumed to be 618 mmolH_2_O m^−2^ s^−1^ as reported by González-Fernández et al. [31]. A second parameterization of this model was defined and tested according to the *g_s_* measurements that were performed on both experiments. The use of two *g_s_* parameterizations allowed the calculation of two different phytotoxic ozone doses (PODs) and the definition of two different critical levels for O_3_ risk assessment based on the generic and local parameterization, respectively.

Once *g_s_* was obtained, the POD was calculated from the O_3_ concentration measured inside the OTCs at flag leaf level by means of the big-leaf resistive scheme described in the MM. This scheme includes also the O_3_ deposition on the external leaf surfaces (cuticles) represented by a resistance to O_3_ deposition R_ext_ of 2500 s/m [26] per unit of vegetal surface (SAI = green + senescent LAI). Synthetically:

(2) $$F_{stom,O_{3}}=O_{3}\times \left(LAI\times g_{s}\times 0.663\right)\times [R_{c}/(R_{b}+R_{c})]$$

where F_stom,O_3__ is the stomatal O_3_ flux, R_c_ is the canopy resistance to the O_3_ deposition, resembling both the O_3_ uptake by stomata and the O_3_ deposition on the external cuticles of leaves (Equation (3)), and R_b_ is the resistance to O_3_ of the sub-laminar layer (Equation (4)).

(3) $$R_{c}=1/(g_{s}\times 0.663\times LAI+SAI/R_{ext})$$

(4) $$R_{b}=1.3\times 150\times \sqrt{d/u}$$

where u is the wind speed inside the OTCs and d is the crosswind leaf dimension (Table 1). The 0.663 value in the Equation (3) represents the diffusivity ratio between O_3_ and water vapor in air [49,50] which is used to convert the *g_s_* to H_2_O into *g_s_* to O_3_. The 1.3 and 150 values are empirical values given by McNaughton and van den Hurk [51] for their R_b_ parameterization. In this work, a unitary LAI and SAI were considered for all the calculations. 

Finally, the phytotoxic ozone dose above the *Y* threshold accounting for the detoxification capacity of plants (POD_Y_) was calculated by integrating the hourly O_3_ stomatal flux $F_{stom,O_{3}}$ (expressed in nmolO_3_ m^−2^ PLA s^−1^) along the accumulation period defined for *T*. *durum* [26] (Equation (5)) with the application of a detoxifying threshold *Y* of 6 nmolO_3_ m^−2^ s^−1^ recommended for both spring wheat and durum wheat by the MM [26] and De La Torre et al. [52], respectively.

(5) $${POD}_{Y}(mmolO_{3}{\;m}^{-2})=\sum \left[\left(F_{stom,O3}-Y\left(nmolO_{3}m^{-2}s^{-1}\right)\right)\times 3600\left(sh^{-1}\right)\times 10^{-6}\right]$$

The AOT40 exposure index was calculated with Equation (6) as the diurnal sum of the hourly exceedances of O_3_ concentration above 40 ppb (considering the hours with solar radiation exceeding 50 Wm^−2^) over the experimental time duration: 


(6) $$AOT40\left(ppm.h\right)=\sum \left({{[O}_{3}]}_{i}-40\right)\times ∆t\;\begin{array}{l} \forall {i:\;\left[O\right]}_{3}>40\\ \forall i:Rad\geq 50\;Wm^{-2} \end{array}$$

### 4.3. Stomatal Conductance Measurements

Measurements of the stomatal conductance to water were performed 4 times during the 2013 season (27 May, 7, 13, and 16 June) and 4 times in 2014 (6, 14, 24 May, and 8 June) on the adaxial surface of flag leaves. Three plants were randomly chosen for each cultivar and OTC and measured with a dynamic diffusion portable porometer (AP4 Delta-T Devices, Cambridge, UK) that was calibrated before each measurement cycle. Three different daily cycles of measurements were performed between 7.00 and 9.00, 12.00 and 14.00, and 16.00 and 18.00 for the morning, midday, and afternoon cycles, respectively. This dataset was used to derive a local parameterization for the *g_s_* model to calculate the phytotoxic ozone dose. The maximum stomatal conductance (*g_max_*) to water was determined as the 90th percentile of all valid stomatal conductance measurements. The limiting functions *f*_Temp_, *f*_Light_, *f*_VPD,_ and *f*_SWC_, which yield a value between 0 and 1, were defined by boundary layer analysis, based on the values corresponding to the 98th percentile of relative stomatal conductance (*g_s_*/*g_max_*) plotted for each class of values of the environmental variables considered (Temp, VPD, SWC, and PAR).

### 4.4. Biomass and Grain Yield Determination

Plants were harvested on 15 July 2013 and 28 June 2014, 128 and 117 days after sowing, respectively (Table 2). All the plants of a single pot were cut and weighted to obtain the aboveground fresh biomass. Then, the total number of spikes per plant was determined. Plants of each pot were then dried in an oven at 80 °C until their weight was constant (at least 72 h), obtaining the aboveground dry biomass. Spikes were then separated from stems and the grain yield was assessed for each single pot by de-hulling the spikes with a laboratory M3B micro-thresher (Co.Mi.L, Rome, Italy). The grain hectolitre weight was determined with a Grain Analyzer Computer 2000 (Dickey-John, Auburn, IL, USA). The harvest index was calculated as the ratio between the grain weight and the aboveground dry biomass per pot.

### 4.5. Determination of the Dose–Response Relationships

For each cultivar, a specific relative effect of O_3_ on grain yield parameters was calculated based on the POD6 values of each OTC. The two cultivars were individually analyzed because in both experiments they showed different phenological developments which accounted for different accumulation times of the POD6. 

The *g_s_* instead was modeled at a general level for *T*. *durum* (no distinction between the two cultivars), according, however, to two different parameterizations: the one suggested by the MM [26] and a local parameterization defined from the *g_s_* measurements performed during the experiments. 

The O_3_ dose–response relationships for aboveground dry biomass, grain yield, and hectolitre weight were obtained by joining the results of the two cultivars collected during the two experiments. The relative effects were calculated as the ratio between the observed mean value of the considered parameter and their calculated values under hypothetical conditions of null POD6 uptake. The latter value was estimated as the intercept of the linear regression of the observed absolute values [53].

### 4.6. Statistical Analysis

The statistical significance of the differences in the crop yield parameters between plants of the different O_3_ treatments was assessed for each cultivar with a one-way analysis of variance (ANOVA) with O_3_ as the fixed factor at two levels in 2013 and at four levels in 2014. The normal distribution of the data of each parameter within each treatment group was verified by the Shapiro–Wilk W Test. The assumption of the homogeneity of the variances was verified for each parameter by the Levene’s test. The Tukey post hoc analysis was used to test the significance of the response of each single O_3_ treatment applied in the 2014 experiment. Significant differences among the means were accepted at *p* < 0.05.

## Figures and Tables

**Table 1:** Comparison between the *g_s_* parameterization for durum wheat defined with the local measurements performed during the experiments (local param.) and the one available in the Mapping Manual [26] (MM param.). For the definition of the parameters, please refer to [26].

Function	Parameter	Unit	Local Param.	MM Param.
	*g_max_*	mmolO^3^ m^−2^ s^−1^	465	410
***f*_min_**		fraction	0.01	0.01
***f*** **_Light_**	a	Adim.	0.0085	0.0105
***f*_Temp_**	T_min_	°C	13	11
	T_opt_	°C	27	28
	T_max_	°C	45	45
***f*_VPD_**	VPD_max_	kPa	2.5	3.1
	VPD_min_	kPa	5.4	4.9
	ΣVPD_crit_	kPa	16	16
***f*_SWC_**	SWC_max_	%vol	19	18
	SWC_min_	%vol	3	4.1
***f*_Phen_**	*f*_Phen_a_	fraction	0.0	0.0
	*f*_Phen_b_	fraction	-	-
	*f*_Phen_c_	fraction	-	-
	*f*_Phen_d_	fraction	-	-
	*f*_Phen_e_	fraction	0.99	0.99
	*f*_Phen_1_ETS_	°C day	−300	−300
	*f*_Phen_2_ETS_	°C day	0	0
	*f*_Phen_3_ETS_	°C day	100	100
	*f*_Phen_4_ETS_	°C day	0	0
	*f*_Phen_5_ETS_	°C day	675	675
	Leaf dimension	cm	2	2

**Table 2:** Main phenological stages of durum wheat plants during the experiments and O_3_ treatment periods. DAS = Days After Sowing.

		2013		2014	
		Colombo	Sculptur	Colombo	Sculptur
Sowing	Date	9 March	9 March	3 March	3 March
Plant Emergence	DAS	12	12	10	10
Plant raising	DAS	48	45	42	42
Start of O_3_ treatments	DAS	58	58	51	51
Flag leaf emergence	DAS	67	64	61	58
Earing	DAS	77	74	72	69
Anthesis	DAS	85	82	78	75
End of O_3_ treatments	DAS	113	113	113	113
Harvest	DAS	128	128	117	117
O_3_ treatment duration	Days	56	56	56	56

**Table 3:** Mean values of POD6 and AOT40 calculated for the two durum wheat cultivars in the different O_3_ treatments in 2013 and 2014 with the MM and the local *g_s_* parameterizations.

	Colombo		Sculptur		
	MM Param.	Local Param.	MM Param.	Local Param.	AOT40
unit	mmolO_3_ m^−2^	mmolO_3_ m^−2^	mmolO_3_ m^−2^	mmolO_3_ m^−2^	ppm.h
**2013**					
CF-OTC	0.80	1.09	0.76	1.05	0.83
OZ++ OTC	11.35	12.66	11.47	12.80	21.12
**2014**					
CF-OTC	1.15	1.50	1.14	1.55	0.96
NF OTC	5.88	6.69	5.96	6.95	6.38
OZ+ OTC	8.39	9.46	8.76	10.03	9.78
OZ++ OTC	15.68	17.31	15.90	17.68	22.60

## Data Availability

Dataset available on request from the authors.

## References

[1] Emberson L. (2020). Effects of ozone on agriculture, forests and grasslands. Philos. Trans. R. Soc. A.

[2] Ramya A., Dhevagi P., Poornima R., Avudainayagam S., Watanabe M., Agathokleous E. (2023). Effect of ozone stress on crop productivity: A threat to food security. Environ. Res..

[3] Montes C.M., Demler H.J., Li S., Martin D.G., Ainsworth E.A. (2022). Approaches to investigate crop responses to ozone pollution: From O_3_-FACE to satellite-enabled modeling. Plant J..

[4] FAOSTAT—Food and Agriculture Organization of the United Nations. http://www.fao.org/faostat/en/#data.

[5] Xu Y., Kobayashi K., Feng Z. (2024). Wheat yield response to elevated O_3_ concentrations differs between the world’s major producing regions. Sci. Total Environ..

[6] Mills G., Sharps K., Simpson D., Pleijel H., Broberg M., Uddling J., Jaramillo F., Davies W.J., Dentener F., Van den Berg M. (2018). Ozone pollution will compromise efforts to increase global wheat production. Global Chang. Biol..

[7] Feng Z., Xu Y., Kobayashi K., Dai L., Zhang T., Agathokleous E., Calatayud V., Paoletti E., Mukherjee A., Agrawal M. (2022). Ozone pollution threatens the production of major staple crops in East Asia. Nat. Food.

[8] Pleijel H., Eriksen A.B., Danielsson H., Bondesson N., Selldén G. (2006). Differential ozone sensitivity in an old and a modern Swedish wheat cultivar—Grain yield and quality, leaf chlorophyll and stomatal conductance. Environ. Exp. Bot..

[9] Monga R., Marzuoli R., Alonso R., Bermejo V., González-Fernández I., Faoro F., Gerosa G. (2015). Varietal screening of ozone sensitivity in Mediterranean durum wheat (*Triticum durum*, Desf.). Atmos. Environ..

[10] Cao J.L., Liang W., Qing Z., Liang J., Hao-Ye T., Zu-Bin X., Gang L., Jian-Guo Z., Kobayashi K. (2009). Characteristics of photosynthesis in wheat cultivars with different sensitivities to ozone under O_3_-free air concentration enrichment conditions. Acta Agron. Sin..

[11] Feng Z., Pang J., Kobayashi K., Zhu J., Ort D.R. (2011). Differential responses in two varieties of winter wheat to elevated ozone concentration under fully open-air field conditions. Glob. Chang. Biol..

[12] Zhu X., Feng Z., Sun T., Liu X., Tang H., Zhu J., Guo W., Kobayashi K. (2011). Effects of elevated ozone concentration on yield of four Chinese cultivars of winter wheat under fully open-air field conditions. Global Chang. Biol..

[13] Bagard M., Jolivet Y., Hasenfratz-Sauder M., Gérard J., Dizengremel P., Le Thiec D. (2015). Ozone exposure and flux-based response functions for photosynthetic traits in wheat, maize and poplar. Environ. Pollut..

[14] Feng Z., Tang H., Uddling J., Pleijel H., Kobayashi K., Zhu J., Oue H., Guo W. (2012). A stomatal ozone flux–response relationship to assess ozone-induced yield loss of winter wheat in subtropical China. Environ. Pollut..

[15] Pleijel H., Danielsson H., Broberg M.C. (2022). Benefits of the Phytotoxic Ozone Dose (POD) index in dose-response functions for wheat yield loss. Atmos. Environ..

[16] Picchi V., Iriti M., Quaroni S., Saracchi M., Viola P., Faoro F. (2010). Climate variations and phenological stages modulate ozone damages in field-grown wheat. A three-year study with eight modern cultivars in Po Valley (Northern Italy). Agric. Ecosyst. Environ..

[17] Reichenauer T.G., Goodman B.A., Kostecki P., Soja G. (1998). Ozone sensitivity in Triticum durum and T. aestivum with respect to leaf injury, photosynthetic activity and free radical content. Physiol Plant..

[18] Herbinger K., Tausz M., Wonisch A., Soja G., Sorger A., Grill D. (2002). Complex interactive effects of drought and ozone stress on the antioxidant defence systems of two wheat cultivars. Plant Physiol. Biochem..

[19] Karlsson P.E., Uddling J., Braun S., Broadmeadow M., Elvira S., Gimeno B.S., Le Thiec D., Oksanen E., Vandermeiren K., Wilkinson M. (2004). New critical levels for ozone effects on young trees based on AOT40 and simulated cumulative leaf uptake of ozone. Atmos. Environ..

[20] Faoro F., Iriti M. (2005). Cell death behind invisible symptoms: Early diagnosis of ozone injury. Biol. Plant..

[21] Evans L.S., Adamski J.H., Renfro J.R. (1996). Relationships between cellular injury, visible injury of leaves, and ozone exposure levels for several dicotyledonous plant species at Great Smoky Mountains National Park. Environ. Exp. Bot..

[22] Biswas D.K., Xu H., Li Y.G., Liu M.Z., Chen Y.H., Sun J.Z., Jiang G.M. (2008). Assessing the genetic relatedness of higher ozone sensitivity of modern wheat to its wild and cultivated progenitors/relatives. J. Exp. Bot..

[23] Gerosa G., Marzuoli R., Rossini M., Panigada C., Meroni M., Colombo R., Faoro F., Iriti M. (2009). A flux-based assessment of the effects of ozone on foliar injury, photosynthesis, and yield of bean (*Phaseolus vulgaris* L. cv. Borlotto Nano Lingua di Fuoco) in open-top chambers. Environ. Pollut..

[24] Fuhrer J., Achermann B. (1999). Critical levels for ozone–level II. Environ. Doc..

[25] Massman W.J., Musselman R.C., Lefohn A.S. (2000). A conceptual ozone dose-response model to develop a standard to protect vegetation. Atmos. Environ..

[26] (2017). LRTAP Convention Chapter 3: Mapping Critical Levels for Vegetation. LRTAP Convention Modelling and Mapping Manual.

[27] Danielsson H., Karlsson P.E., Pleijel H. (2013). An ozone response relationship for four *Phleum pratense* genotypes based on modelling of the phytotoxic ozone dose (POD). Environ. Exp. Bot..

[28] Pleijel H., Danielsson H., Emberson L., Ashmore M.R., Mills G. (2007). Ozone risk assessment for agricultural crops in Europe: Further development of stomatal flux and flux–response relationships for European wheat and potato. Atmos. Environ..

[29] Emberson L.D., Ashmore M.R., Cambridge H.M., Simpson D., Tuovinen J. (2000). Modelling stomatal ozone flux across Europe. Environ. Pollut..

[30] Mediavilla S., Escudero A. (2004). Stomatal responses to drought of mature trees and seedlings of two co-occurring Mediterranean oaks. For. Ecol. Manag..

[31] González-Fernández I., Bermejo V., Elvira S., De La Torre D., González A., Navarrete L., Sanz J., Calvete H., García-Gómez H., López A. (2013). Modelling ozone stomatal flux of wheat under mediterranean conditions. Atmos. Environ..

[32] Tomer R., Bhatia A., Kumar V., Kumar A., Singh R., Singh B., Singh S.D. (2015). Impact of Elevated Ozone on Growth, Yield and Nutritional Quality of Two Wheat Species in Northern India. Aerosol Air Qual. Res..

[33] Gerosa G., Marzuoli R., Finco A., Monga R., Fusaro I., Faoro F. (2014). Contrasting effects of water salinity and ozone concentration on two cultivars of durum wheat (*Triticum durum* Desf.) in Mediterranean conditions. Environ. Pollut..

[34] Mashaheet A.M., Burkey K.O., Saitanis C.J., Abdelrhim A.S., Rafiullah, Marshall D.S. (2020). Differential ozone responses identified among key rust-susceptible wheat genotypes. Agronomy.

[35] Ma L., Chen C., Cotrozzi L., Bu C., Luo J., Yao G., Chen G., Zhang W., Nali C., Lorenzini G. (2022). The effects of elevated tropospheric ozone on carbon fixation and stable isotopic signatures of durum wheat cultivars with different biomass and yield stability. Plants.

[36] Picchi V., Risoli S., Nali C., Lorenzini G., D’Asaro L., Pisuttu C., Pellegrini E. (2023). Identification of key responsive leaf physiochemical traits for ozone sensitivity in 16 Italian cultivars of Triticum durum subjected to peak concentrations. J. Agron. Crop Sci..

[37] Picchi V., Monga R., Marzuoli R., Gerosa G., Faoro F. (2017). The ozone-like syndrome in durum wheat (*Triticum durum* Desf.): Mechanisms underlying the different symptomatic responses of two sensitive cultivars. Plant Physiol. Biochem..

[38] Barnes J., Bender J., Lyons T., Borland A. (1999). Natural and man-made selection for air pollution resistance. J. Exp. Bot..

[39] Marzuoli R., Gerosa G., Bussotti F., Pollastrini M. (2019). Assessing the impact of ozone on forest trees in an integrative perspective: Are foliar visible symptoms suitable predictors for growth reduction? A critical review. Forests.

[40] Rai R., Agrawal M., Agrawal S.B. (2007). Assessment of yield losses in tropical wheat using open top chambers. Atmos. Environ..

[41] EUROSTAT—Crop Production in EU Standard Humidity [apro_cpsh1]. https://ec.europa.eu/eurostat/data/database.

[42] ISTAT—Estimation of the Area and Production of Agricultural and Floral Cultivation, and Pot Plants. http://dati.istat.it/Index.aspx?QueryId=33702#.

[43] Grünhage L., Pleijel H., Mills G., Bender J., Danielsson H., Lehmann Y., Castell J., Bethenod O. (2012). Updated stomatal flux and flux-effect models for wheat for quantifying effects of ozone on grain yield, grain mass and protein yield. Environ. Pollut..

[44] (1998). Durum Wheat Grains. Qualitative Requirements, Classification and Test Methods.

[45] Marzuoli R., Monga R., Finco A., Gerosa G. (2016). Biomass and physiological responses of *Quercus robur* (L.) young trees during 2 years of treatments with different levels of ozone and nitrogen wet deposition. Trees—Struct. Funct..

[46] Pleijel H. (2011). Reduced ozone by air filtration consistently improved grain yield in wheat. Environ. Pollut..

[47] Olszyk D.M., Bytnerowicz A., Takemoto B.K. (1989). Photochemical oxidant pollution and vegetation: Effects of mixtures of gases, fog and particles. Environ. Pollut..

[48] Agathokleous E., Araminiene V., Belz R.G., Calatayud V., De Marco A., Domingos M., Feng Z., Hoshika Y., Kitao M., Koike T. (2019). A quantitative assessment of hormetic responses of plants to ozone. Environ. Res..

[49] Massman W.J. (1998). A review of the molecular diffusivities of H_2_O, CO_2_, CH_4_, CO, O_3_, SO_2_, NH_3_, N_2_O, NO, and NO_2_ in air, O_2_ and N_2_ near STP. Atmos. Environ..

[50] Nobel P.S. (1999). Physicochemical & Environmental Plant Physiology.

[51] McNaughton K.G., Van den Hurk B. (1995). A ‘Lagrangian’revision of the resistors in the two-layer model for calculating the energy budget of a plant canopy. Bound.-Layer Meteorol..

[52] De la Torre D. (2010). Relative yield loss calculations in wheat (*Triticum durum* Desf. cv. Camacho) due to ozone exposure. Sci. World J..

[53] Fuhrer J. (1994). The Critical Level for Ozone to Protect Agricultural Crops—An Assessment of Data from European Open-Top Chamber Experiments, Critical Levels for Ozone: A UN-ECE Workshop Report.

